# Evaluation of antimycobacterial activity of *Curcuma xanthorrhiza* ethanolic extract against *Mycobacterium tuberculosis* H37Rv *in vitro*

**DOI:** 10.14202/vetworld.2018.368-372

**Published:** 2018-03-27

**Authors:** Ngadino, Setiawan, Koerniasari, Ernawati, S. A. Sudjarwo

**Affiliations:** 1 Study Program of Environmental Health, Polytechnic of Health, Surabaya, Indonesia; 2 Department of Pharmacology, Faculty of Medicine, Wijaya Kusuma University, Surabaya, Indonesia; 3 Department of Pharmacology, Faculty of Veterinary Medicine, Airlangga University, Surabaya, Indonesia

**Keywords:** antimycobacterial, Curcuma xanthorrhiza, minimal bactericidal concentration, minimal inhibitory concentration

## Abstract

**Aim:**

The aim of this study was to evaluate the antimycobacterial activity of the *Curcuma xanthorrhiza* ethanolic extract *in vitro*.

**Materials and Methods:**

Ethanolic extract of *C. xanthorrhiza* was set by maceration method. The broth microdilution and disc diffusion method were used to determine the minimal inhibitory concentration (MIC) and minimal bactericidal concentration (MBC), respectively, of *C. xanthorrhiza* ethanol extract on strain *Mycobacterium tuberculosis* H37Rv.

**Results:**

*C. xanthorrhiza* ethanol extract was found to have the antimycobacterial effects with a MIC value of 1600 μg/ml while MBC value of 3200 μg/ml for *M. tuberculosis* H37Rv.

**Conclusion:**

From these findings , it can be concluded that *C. xanthorrhiz*a ethanol extract have an antibacterial activity against *Mycobacterium tuberculosi*s H37Rv *in vitr*o and its potency elevated by increasing the *C. xanthorrhiz*a ethanol extract concentration.

## Introduction

Tuberculosis (TB) is a serious public health problem, one of the leading causes of mortality worldwide, infecting about 9 million people, kills approximately 2 million people annually. The global incidence rate for TB is growing each year by approximately 1.1% and the number of cases by about 2.4%. Resistance to anti-TB drugs continued to be recognized as a clinical problem through the latter part of the 21^st^ century. As a result, multidrug-resistant and extensively drug-resistant TB are now becoming a major threat to health worldwide, accounting for almost 3% of all newly reported cases of TB [1]. Due to increased drug-resistant strains of bacteria such as *Mycobacterium tuberculosis* and methicillin-resistant *Staphylococcus aureus*, there has been renewed interest in herbal as potential sources of novel antibiotics. The World Health Organization estimated that annual global use of herbal medicines is about US $83 billion in 2008, indicating that natural products are important sources of new therapeutics and future medicines [1].

The use of herbal as medicine is well known in rural areas of many developing countries. Most herbal medicines are well tolerated by the patient, with fewer unintended consequences than synthetic medicine. Herbs typically have fewer side effects than synthetic medicine, and may be safer to use over time [2,3]. The findings of the new antibacterial compounds in herbal became one of the remarkable alternatives for treatments since they are rich in numerous varieties of secondary metabolites such as alkaloids, flavonoids, tannins, saponin, and phenolic compounds with antibacterial properties [4]. Medicinal plant products have long been used as antibacterial in traditional medicines, for the treatment of many diseases such as TB. The anti-*M. tuberculosis* of medicinal plant products has become subject to scientific investigations currently worldwide, and their active components provide a potential alternative to conventional anti-*M. tuberculosis*. In this context, the development of medicinal plant product-based drug candidates as anti-*M. tuberculosis* has gained momentum in research studies directed toward design and discovery of drugs [5]. *Piliostigma thonningii* [5], *Curtisia dentata* [6]*, Combretum zeyheri* [7], *Artemisia nilagirica*, and *Murraya koenigii* [8] are among the medicinal plants claimed to possess potential antimycobacterial agent.

It has been demonstrated that the medicinal properties of *Curcuma xanthorrhiza* were due to the phytochemicals possessed including alkaloids, phenols, flavonoids, triterpenes, sterols, glycosides, and terpenoids. *C. xanthorrhiz*a contain xanthorrhizol and curcumin may be used for antioxidant and anticancer [9], antibacterial [10-12], antiviral and antifungal [13], endothelial cell protection on hypercholesterolemia [14], pancreas protective on methylmercury toxicity [15], and testicular protective on lead acetate toxicity [3].

Recent research activities have shown that *C. xanthorrhiza* belongs to the family *Zingiberaceae* and the rhizome extract contains active phytochemical constituents with xanthorrhizol and curcumin as the main compounds [11,16,17]. Xanthorrhizol and curcumin isolated from the ethanolic rhizome extract of *C. xanthorrhiza* show potent antibacterial activity against a wide spectrum of Gram-positive and negative bacterial pathogen [9,14]. It also has been reported that xanthorrhizol showed the highest antibacterial activity against *Escherichia coli*, *Propionibacterium acnes*, *Streptococcus mutans*, *Actinomyces viscosus, Porphyromonas gingivalis*, *S*. *aureus*, *Klebsiella pneumoniae, Pseudomonas* spp., and *Bacillus cereus* [9,11], while curcumin also showed effective against *Salmonella typhimurium, Pseudomonas aeruginosa, E. coli, S. aureus*, *B. cereus, Helicobacter pylori*, and *Listeria monocytogenes* [16,17,18].

*C. xanthorrhiza* has drawn the attention of researchers because of their suitable applications in the fields of material science and medicine. Therefore, the objective of the present study was to evaluate the antimycobacterial activity of the *C. xanthorrhiza* ethanol extract *in vitro*.

## Materials and Methods

### Ethical approval

The study was conducted in the Department of Veterinary Pharmacology, Faculty of Veterinary Medicine, Airlangga University. All procedure employed was approved by the Ethical Clearance Committee for preclinical research, Institute of Tropical Disease, Airlangga University.

### Preparation of ethanol extract of *C. xanthorrhiza*

Plant material and extract preparation of *C. xanthorrhiza* were collected from Surabaya, Indonesia. *C. xanthorrhiza* materials were cleaned with running tap water and chopped into pieces. They were dried under shade at ambient temperature for 5 days, and the air dried *C*. *xanthorrhiza* was then ground to powder for extraction. The powdered *C. xanthorrhiza* (1 kg) was macerated with ethanol 96% (5 L) for 1 week at 37°C. The supernatant was then collected and filtered through Whatman No. 1 filter paper in a Buchner funnel under vacuum. The filtrate was concentrated by evaporation with a vacuum rotary evaporator at 45°C [8]. Furthermore, the extract was freeze-dried to get ethanol-free powder.

### Qualitative phytochemical testing

The *C. xanthorrhiza* ethanol extract was subjected to the qualitative phytochemical screening for the presence of some chemical constituents. In the most active extracts, qualitative tests for terpenoids, saponins, tannins, flavonoid, phenols, and alkaloids were carried out as described by Jyoti and Rajeshwari [4].

### Culture and preparation of *M. tuberculosis*

*M. tuberculosis* strains H37Rv were obtained from the Institute of Tropical Disease, Airlangga University, Surabaya, Indonesia. *M. tuberculosis* was cultured at 37°C in Middlebrook 7H9 broth (Becton Dickinson, Sparks, MD) supplemented with 0.2% glycerol (Sigma Chemical Co., St. Louis, MO) and 10% oleic acid albumin dextrose catalase (OADC; Becton Dickinson) until logarithmic growth was reached. Each culture was mixed with a sufficient volume of sterile supplemented Middlebrook 7H9 broth to achieve a turbidity equivalent to that of McFarland’s No. 1 standard. To obtain the test inoculum, this suspension was further diluted 1:50 with the same culture medium to approximately 6×10^6^ colony-forming units (CFU)/mL immediately before use [19].

### Minimum inhibitory concentration (MIC) determination by resazurin microtiter plate assay (REMA) method

REMA was performed with minor modifications [20]. The REMA plate method was performed in 7H9-S medium containing Middlebrook broth, 0.1% Casitone, and 0.5% glycerol and supplemented with OADC (Becton-Dickinson). Briefly, 100 μL of Middlebrook 7H9 broth was dispensed into each well of the microtiter plate. Serial two-fold dilutions of *C. xanthorrhiz*a extract (powder-free ethanol) were performed in Middlebrook 7H9 broth to obtain final drugs concentration 0; 200; 400; 800; 1600, and 3200 μg/ml, and rifampicin (10 μg/ml) was used as a standard drug and were made in the plate. *M. tuberculosis* strains H37Rv suspension (100 μL) containing approximately 6×10^6^ CFU/mL were added to all the wells. Sterility control and growth control were also included. The plate was wrapped in aluminum foil and incubated at 37°C for 7 days. After completion of the incubation period, 30 μL resazurin solution (100 μg/mL) was added to each well and plate was again wrapped in aluminum foil and incubated overnight. The plate was then observed for change in color. The color change from blue to pink or colorless indicated the growth of the bacteria. The lowest concentration of *C. xanthorrhiza* ethanolic extract that prevented color a change from blue to pink was taken as the upper limit for MIC range, and the highest *C. xanthorrhiza* ethanolic extract concentration that showed a change in color from blue to pink was considered the lower limit. All evaluations were carried out in quadruplicate.

### Minimum bactericidal concentration (MBC) determination using the paper disc diffusion method

Screening of *C. xanthorrhiza* ethanolic extract and its solvents for antimycobacterial activity against *M. tuberculosis* strain *H37Rv* was done using the paper disc diffusion method [18]. Serial two-fold dilutions of *C. xanthorrhiza* extract (powder-free ethanol) were performed in a distilled water solution (0, 200, 400, 800, 1600, and 3200 μg/ml) and were slowly absorbed into the sterilized paper disc (diameter: 8 mm, Watman, England) and adhered to the surface of the plate on which *M. tuberculosis* strains H37Rv at a concentration of 10^6^ CFU/ml had been inoculated in Middlebrook 7H9 broth. Sterilized distilled water was used as a control. After culturing for 24 h in an incubator at 370C, antibacterial activity was defined as the diameter (mm) of the clear inhibitory zone formed around the discs. MBC was defined as the lowest concentration that induced the clear inhibitory zone formed around the discs [19].

## Results

### Phytochemical of *C. xanthorrhiza* ethanolic extract

The preliminary phytochemical analysis of *C. xanthorrhiza* ethanol extract (Table-1) showed the presence of alkaloids, saponins, flavonoids, triterpenoids, and tannins of phytochemicals. Any of these phytochemicals, either singly or in a combination with each other could be responsible for the antibacterial activity of the *C. xanthorrhiza* ethanol extract.

**Table-1:** Phytochemical analysis of extracts of *Curcuma xanthorrhiza* ethanol extract.

*Curcuma xanthorrhiza* ethanol extract	

Phytochemical	Level
Alkaloids	++
Phenols	+++
Saponins	++
Flavonoids	++
Triterpenoids	+++
Tannins	

+: Low, ++: Immediate, +++: High

### The MIC of the *C. xanthorrhiza* ethanol extract against M. tuberculosis on REMA method

**Figure-1:**
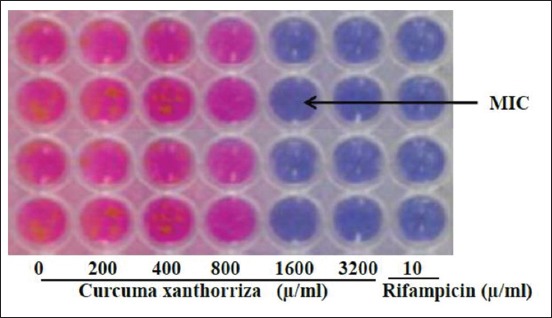
Minimal inhibitory concentration of *Curcuma xanthorrhiza* ethanol extract against *Mycobacterium tuberculosis* H37Rv on resazurin microtiter plate assay method was 1600 μg/ml (Black arrows). Serial two-fold dilutions *C. xanthorrhiza* ethanol extract (0 μg/ml, 200 μg/ml, 400 μg/ml, 800 μg/ml, 1600 μg/ml, and 3200 μg/ml) and rifampicin (10 μg/ml).

The MIC of the *C. xanthorrhiza* ethanolic extract was determined for their antimycobacterial activity using resazurin as an indicator of *M. tuberculosis* viability in 96-well microplates. The investigation showed that *C. xanthorrhiza* ethanolic extract was active against *M. tuberculosis*. In this study, MIC of *C. xanthorrhiza* ethanol extract against *M. tuberculosis* strains H37Rv was 1600 μg/mL, while rifampicin was 10 μg/mL (Figure-1).

### The MBC of the *C. xanthorrhiza* ethanolic extract against M. tuberculosis

**Figure-2:**
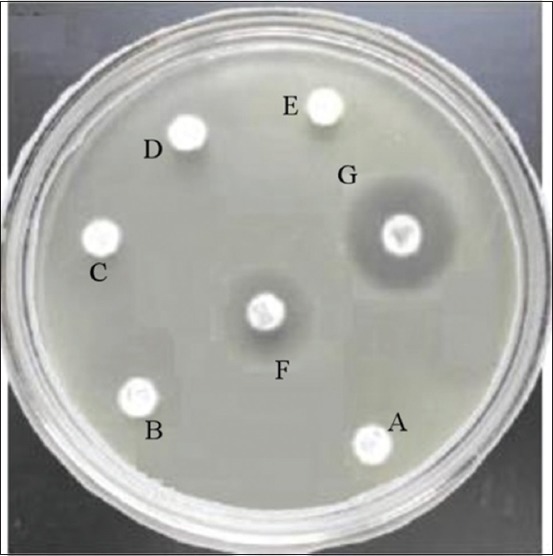
Minimal bactericidal concentration of *Curcuma xanthorrhiza* ethanol extract against *M. tuberculosis* H37Rv on the plates of middle brook 7H9 broth was 3200 μg/ml. *C. xanthorrhiza* ethanol extract at dose 0 μg/ml (a), 200 μg/ml (b), 400 μg/ml (c), 800 μg/ml (d), 1600 μg/ml (e), and 3200 μg/ml (f) and rifampicin 10 μg/ml (g).

The antimycobacterial activity of *C. xanthorrhiza* ethanolic extract against *M. tuberculosis* strains H37Rv was done using the plates of Middle brook 7H9 broth. MBC was defined as the lowest concentration that produced no growth *M. tuberculosis* strains H37Rv at Middle brook 7H9 broth. The MBC *C. xanthorrhiza* ethanolic extract against *M. tuberculosis* strains H37Rv was 3200 μg/mL, which produced no growth *M. tuberculosis* strains H37Rv at Middle brook 7H9 broth (Figure-2).

## Discussion

TB is a chronic disease caused by *M. tuberculosis*. The emergence of antibiotic-resistant strains of this species underscores the need for novel effective drugs against resistant mycobacteria as first-line anti-TB medications [6]. The uses of natural product as traditional medicines are accepted, particularly in developing countries. Medicinal plants have been used for centuries as remedies for human diseases because they contain components of therapeutic value [2]. Extraction of bioactive compounds from medicinal plants permits the demonstration of their physiological activity. It also facilitates pharmacology study leading to the synthesis of more potent drugs for meeting demand for effective and safe use [8]. This led us to the investigation of the effects of *C. xanthorrhiza* ethanolic extract on antimycobacterial activity. Phytochemical analysis of *C. xanthorrhiza* ethanolic extract showing antibacterial activity revealed *C. xanthorrhiza* ethanolic extract contained alkaloids, tannin, flavonoid, phenol, and saponin. There are reports showing that phenol, alkaloids, and flavonoids are the responsible compounds for the antibacterial activities in higher plants [17]. *In vitro* and *in vivo* toxicity shown that the *C. xanthorrhiza* extract non-toxic to peripheral blood mononuclear cells and in mice (data not shown).

The MIC is defined as the lowest concentration of an antimicrobial agent that prevents the visible growth of test microorganism in the 96-well microtiter plates. The first well with no visible growth after the incubation period was taken as the MIC. The MBC is the minimal concentration of the antimicrobial that kills the inoculums and can be determined by broth dilution MIC test [19].

Investigation of MIC and MBC plays an important role during the process of screening, prioritizing, and optimizing a chemical series during early antibacterial drug discovery. The MIC of the *C. xanthorrhiz*a ethanolic extract was determined for their antimycobacterial activity using REMA method. Many researchers have used the REMA method to screen test substances for antimycobacterial activity against *M. tuberculosis*. Resazurin, an oxidation-reduction indicator, has been used to assess viability and bacterial contamination and to test for antimicrobial activity. Results obtained using the REMA assay is faster and less expensive. Bearing in mind considerations of rapidity, low technology requirements, and low cost, microplate assays that use resazurin type compounds have the potential of becoming the methods of choice for drug susceptibility testing of *M. tuberculosis* in places where TB is a major problem [20]. The MBC of the *C. xanthorrhiza* ethanol extract against *M. tuberculosis* was conducted on the plates of Middle brook 7H9 broth. In this study, MIC of *C. xanthorrhiza* ethanol extract against *M. tuberculosis* strains H37Rv was 1600 μg/mL, while MBC of *C. xanthorrhiza* ethanol extract against *M. tuberculosis* strains H37Rv was 3200 μg/mL. This suggests that *C. xanthorrhiza* ethanol extract has potent activity as antimycobacterial and this has been confirmed experimentally. These results are in agreement with reports in the literature that have documented the antibacterial activity of *C. xanthorrhiza* ethanol extract against a large number of Gram-positive and Gram-negative bacteria [10,17]. Some research has also shown that *C. xanthorrhiza* ethanol extract generally showed stronger effects for Gram-positive bacteria (e.g., *Streptococcus mutans*, *L. monocytogenes, Bacillus megaterium, B. cereus*, *S. aureus, Staphylococcus epidermidis*, and *Lactobacillus plantarum*) and for Gram-negative bacteria (e.g., *E. coli, Pseudomonas fluorescens, P. aeruginosa, Salmonella typhimurium, Vibrio parahaemolyticus*, and *Klebsiella pneumoniae*) [11-13,16,17]. Previous studies have shown that phenolic compounds of *C. xanthorrhiza* may act on microbial cell walls or membranes. They inhibit microbial growth by changing microbial cell permeability, which leads to the loss of intracellular molecules such as protein, DNA, RNA, and ATP [12]. Phenolic compounds could also affect cellular wall, membrane integrity, and microbial physiological responses [18].

Recent research activities have shown that *C. xanthorrhiza* extract contains active phytochemical constituents with xanthorrhizol and curcumin as the main compounds [17]. It has been demonstrated that the antibacterial activity of curcumin against *Bacillus subtilis* occurs through the inhibition of bacterial cell proliferation by blocking the assembly dynamics of FtsZ in the Z ring [18] and caused membrane damage of Gram-positive (*S. aureus* and *Enterococcus faecalis*) and Gram-negative (*E. coli* and *P. aeruginosa*) [12]. While xanthorrhizol strongly inhibited the growth of *B. cereus*, *C. perfringens*, *L. monocytogenes*, *S. aureus*, *Salmonella* Typhimurium, and *V. parahaemolyticus* with MICs of 8, 16, 8, 8, 16, and 8 µg/ml, respectively. These strains were killed by xanthorrhizol at MBCs of 16, 32, 16, 16, 16, and 16 µg/ml, respectively [9-11].

The potency of the extracts of *C. xanthorrhiza* as compared to the standard antimycobacterial drugs (rifampicin) used was still very low. This is understandable considering the fact that rifampicin was pure compounds known to be the most active against *M. tuberculosis* compared with the activity of *C. xanthorrhiza* that was crude extracts. If the active compounds could be isolated and purified of *C. xanthorrhiza*, it is possible that they could show a comparable activity or even more activity than some of the antimycobacterial drugs currently in use.

## Conclusion

Thus, from the results obtained, it can be concluded that *C. xanthorrhiza* ethanol extract has promising anti-TB activity by preliminary *in vitro* techniques. Therefore, it has the definite potential as a source of compounds that may be developed further into antimycobacterial drugs.

## Authors’ Contributions

N, S, K, and E equally contributed in conception and design of the experiment, data collection, data analysis, and interpretation. SAS helped in critical revision of the article and final approval of the version to be published. All the authors read and approved of the final manuscript.
